# Optimization of a 100% Product Utilization Process for LPG Separation Based on Distillation-Membrane Technology

**DOI:** 10.3390/membranes16010040

**Published:** 2026-01-10

**Authors:** Peigen Zhou, Tong Jing, Jianlong Dai, Jinzhi Li, Zhuan Yi, Wentao Yan, Yong Zhou

**Affiliations:** 1 College of Chemical Engineering, Zhejiang University of Technology, Hangzhou 310014, China; 211123010057@zjut.edu.cn (P.Z.);; 2 Sinopec Research Institute of Petroleum Processing Co., SINOPEC, Beijing 100083, China

**Keywords:** full utilization, process design, distillation—membrane separation process design, systematic simulation

## Abstract

This study presents the techno-economic optimization of a hybrid distillation-membrane process for the complete fractionation of liquefied petroleum gas (LPG), targeting high-purity propane, n-butane, and isobutane recovery. The process employs an initial distillation column to separate propane (99% purity) from a propane-enriched stream, which is subsequently fed to a two-stage membrane system using an MFI zeolite hollow-fiber membrane for n-butane/isobutane separation. Through systematic simulation and sensitivity analysis, different membrane configurations were evaluated. The two-stage process with a partial residue-side reflux configuration demonstrated superior economic performance, achieving a total operating cost of 31.58 USD/h. Key membrane parameters—area, permeance, and separation factor—were optimized to balance separation efficiency with energy consumption and cost. The analysis identified an optimal configuration: a membrane area of 800 m^2^, an n-butane permeance of 0.9 kg·m^−2^·h^−1^, and a separation factor of 40. This setup ensured high n-alkane recovery while effectively minimizing energy use and capital investment. The study concludes that the optimized distillation-membrane hybrid process offers a highly efficient and economically viable strategy for the full utilization of LPG components.

## 1. Introduction

With increasing people developing industrialization, the petrochemical industry plays a more important role in the life [1]. Liquefied petroleum gas (LPG) is obtained as a by-product in the petrochemical industry and serves as an important feedstock, rich in high-value light hydrocarbon components such as propane, n-butane (n-C_4_), and isobutane (i-C_4_) [2,3]. These are important industrial chemicals, each serving different functions in various applications [4]. For example, n-butane is widely employed as a feedstock in the manufacture of synthetic rubber, plastics, and other petrochemical derivatives [5]. In contrast, isobutane is commonly used as a refrigerant in cooling systems and as a propellant in aerosol products [6]. Therefore, it is important to recover n-C4 and i-C_4_ from LPG [7]. However, efficient separation of butane isomers remains challenging due to their closely matched physical and chemical properties, such as boiling point [8], kinetic diameter [9], and saturated vapor pressure [10]. Therefore, it is essential to develop a process for complete fractionation and recovery of all LPG components.

At present, various fully separating and recovering processes have been designed. Among them, distillation has attracted widespread attention from scholars due to its wide applicability, technical maturity, and superior continuous operability [11]. For example, Yang et al. recovered tetrafluoroethylene monomer with 99.999% purity from cracked gas by distillation process [12]. Zhou et al. improved cellulose content and reduced sugar yield by hydrothermal pretreatment [13]. Although distillation is capable of producing high-purity products, it is hampered by inherent drawbacks, including high energy consumption [14] and limited separation selectivity [15], which restrict its broader application. In response to the high energy consumption of distillation, researchers have explored the integration of distillation with other separation methods to develop novel and comprehensive recovery processes. He et al. achieved synergistic and efficient removal of multiple impurities by proposing an integrated refining approach [16]. Among these hybrid configurations, the extractive distillation process has gained widespread application in gas separation and recovery due to its straightforward operation [17], high operational flexibility [18], and low energy consumption [19]. For example, Yang et al. separated and recovered isopropyl alcohol and isooctane from industrial wastewater of fuel oil by extractive distillation, with a reduction in total energy consumption of 5% compared to the traditional distillation process [20]. However, extractive distillation suffers from operational difficulties, such as challenging solvent selection [21], process complexity [22], and equipment corrosion [23], which collectively restrict its wider industrial use. The newly emerged membrane separation offers the advantages of compact structure [24], high energy efficiency [25], no environmental pollution [26] and instant dynamic response [27] showing promising prospects to overcome these limits. For example, Liu et al. recovered H_2_ from the H_2_ and CH_4_ by carbon molecular sieve which had a H_2_ permeance of 57.6 GPU and a separation factor of H_2_/CH_4_ of 275.8 [28]. In addition, Fu et al. used an integrated process combining distillation with a NaA zeolite membrane to recover ethanol from an ethanol/water mixture [29]. This approach achieved complete ethanol recovery and achieved zero liquid discharge. Liu et al. prepared ultrathin (27 nm) highly porous all-aromatic polyamide nanofiltration membranes by interfacial polymerization, successfully scaled up to a 2 m^2^ spiral wound module, demonstrating the potential industrial scalability potential [30]. However, the study on the recovery of n-C_4_ and i-C_4_ from LPG by coupling membrane and distillation process is still exiting some shortcoming. Distillation processes are commonly afflicted with the drawbacks of excessive energy consumption and cumbersome operation, while extractive distillation is confronted with intractable issues including the rigorous requirements for solvent selection and elevated process costs. Therefore, developing an innovative process flow with advantages such as simplified separation routes, simple operation, low cost, and low energy consumption holds practical and theoretical value for related separation technologies. Research by Antonia Rom et al. demonstrates that integrating membrane technology with distillation can reduce energy consumption per unit by up to 50% [31].

In this study primarily aims to design a universal full-recovery product process by developing a dedicated process for the complete recovery of products in the LPG separation process. We employ Aspen software v14.0 to simulate and analyze the energy consumption of a hybrid process that integrates distillation with membrane separation for the recovery of n-C_4_ and i-C_4_ from LPG. The configured system utilizes an MFI zeolite hollow fiber membrane, selected for its uniform pore structure (0.5 nm aperture) and robust thermal/chemical stability [32]. For example, Yu et al. synthesized MFI hollow fiber membranes via mild gel recycling, achieving a permeation flux of 1.9 × 10^−7^ mol/(m^2^ s Pa) for n-butane at 303 K and 166 kPa, with a separation factor of 34 for n-isobutane [30]. Furthermore, the process flow and key operating parameters of this hybrid system are systematically evaluated, focusing on their impact on overall energy consumption, separation efficiency, and economic performance. The findings are expected to provide an optimized strategy with promising industrial applicability for the recovery of these C4 isomers from LPG.

## 2. Modeling

### 2.1. Membrane Module Modeling

**Figure 1:**
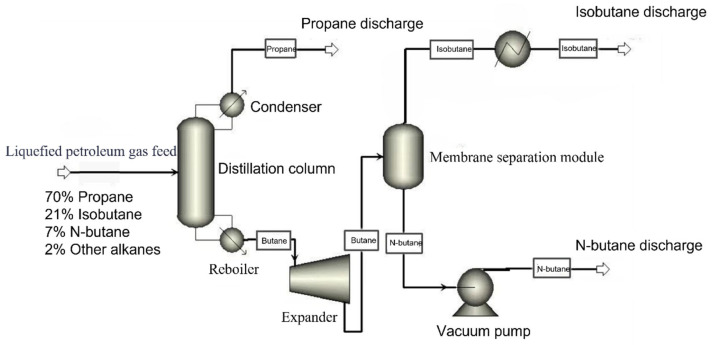
Process Flow Diagram for Separation of Mixed Alkanes.

According to the literature, MFI hollow-fiber membranes with well-defined gas transport channels exhibit promising performance in the separation of n-butane and i-butane [33]. Typical n-butane permeance values range from 0.6 to 1.2 kg·m^−2^·h^−1^, with n-butane/i-butane separation factors generally between 10 and 60 [33,34,35,36,37]. To systematically investigate the performance and economic feasibility of the membrane separation process under various operating conditions, this study develops a mathematical model of the membrane module and conducts numerical simulations using MATLAB R2019b, The specific process is illustrated in Figure 1.

The process design employs fixed reference parameters, such as feed temperature, flow rate, and n-butane recovery rate (≥80%). The study focuses on analyzing the impact of membrane area and permeate flux on energy consumption and total costs under different process configurations.

The process flow in this study assumes a liquefied petroleum gas (LPG) feed flow rate of 1000 m^3^/h, with a composition of 70% propane, 28% butane and 2% other alkanes. Within the butane fraction, n-butane accounts for 30% and i-butane for 70%. The feed temperature is maintained constant at 330 K. Key operating and membrane performance parameters are systematically varied in the simulations: membrane area ranges from 100 to 1500 m^2^, n-butane permeance varies between 0.6 and 1.2 kg·m^2^·h^−1^, and the separation factor spans from 10 to 65. The membrane area (100–1500 m^2^) supports scalable design requirements, ranging from pilot-scale to large industrial installations. The permeate flux (0.6–1.2 kg·m^−2^·h^−1^) balances the properties of MFI membrane materials with operating pressure to optimize efficiency while minimizing fouling [36,37,38]. The separation factor range (10–65) is based on design parameters reported in the current literature [33,34,35,36,37,39,40,41,42]. Permeate-side operating conditions cover compressor outlet pressures of 1–5 kPa and vacuum pump outlet pressures of 0.5–1 kPa.

The separation factor is calculated as follows:

(1) $$\alpha =\frac{Y_{a}/Y_{b}}{X_{a}/X_{b}}$$

In the equation, X and Y represent the mass fractions of each component on the feed side and permeate side, respectively, while a and b denote the distinct gas components.

### 2.2. Energy Consumption Formula

Within the MATLAB computational framework, the energy consumption of the vacuum pump and compressor was determined by the following equations [43]:

(2) $$W=\frac{F}{\eta }\times \frac{\gamma \times R_{g}\times T}{\gamma -1}\times [\varphi_{k}^{\frac{\gamma -1}{\gamma }}-1]$$

In the equations, F represents the inlet flow rate in kmol/s, η denotes the pump efficiency, taken as 85% in this study, and T is the operating temperature. The variable $R_{g}$ refers to the ideal gas constant, while γ represents the specific heat ratio of the gas mixture. For the butane mixture considered here, γ is set to 2.5. The parameter φ defines the pressure ratio, calculated as the ratio of the high-pressure side to the low-pressure side.

The energy consumption of the expander is calculated as follows [43]:

(3) $$W_{p}=\frac{1}{\eta }\times \frac{k}{k-1}\times m\times R\times T\times \left[{\left(\frac{P_{2}}{P_{1}}\right)}^{\frac{k-1}{k}}-1\right]$$

In the equation, η denotes the compressor efficiency, which is set at 80% in this study; k represents the adiabatic index, taken as 1.07; and m is the mass flow rate in kg/s. The parameter R corresponds to the gas constant in J/(kg·K), while P_1_ and P_2_ refer to the inlet pressure and outlet pressure, respectively.

The energy consumption of the distillation column is primarily attributed to the condenser and the reboiler, which can be calculated using the following equations [44]:

(4) $$Q_{\mathrm{reb}}=D\times H_{d}+W\times H_{w}+Q_{\mathrm{cond}}-F\times H_{f}$$

The cost of heating steam is calculated as the product of heating steam consumption and its unit price. The steam consumption is derived from the heat load divided by the latent heat of vaporization of the heating steam. In the equations, $Q_{\mathrm{reb}}$ and $Q_{\mathrm{cond}}$ represent the heat loads of the reboiler and condenser, respectively; R denotes the reflux ratio, specified as 2.5 in this study; and D refers to the mass flow rate of the distillate, set at 30.77 kmol/h. The variables $H_{d}$, $H_{w}$, $H_{f}$ and correspond to the molar enthalpy of the distillate, bottom product, and feed stream, respectively, while F indicates the feed flow rate, maintained at 40.75 kmol/h throughout the simulation [44].

(5) $$Q_{\mathrm{cond}}=\left(R+1\right)\times D\times \lambda$$

(6) $$L’=\frac{Q_{\mathrm{cond}}}{\Delta H}$$

In the equations, $Q_{\mathrm{cond}}$ represents the heat load of the condenser, R denotes the reflux ratio (specified as 2.5 in this study) [45], D refers to the mass flow rate of the distillate (set at 30.77 kmol/h), and ΔH indicates the total latent heat of vaporization of propane, taken as 19,000 kJ/kmol. The total energy consumption of the distillation column is calculated as the sum of the cooling water cost and the heating steam cost.

### 2.3. Cost Formula

Each configuration in the membrane process employs its own cost calculation methodology. The capital cost of the membrane equipment is categorized into two components: the membrane material cost and the membrane module cost. The fixed cost associated with the membrane itself is calculated as follows [46]:

(7) $$C_{1}=\frac{\left(P_{m}+P_{mf}\right)\times A_{m}}{10\times 24\times 365}$$

The fixed membrane cost $C_{1}$ (USD/h) is calculated by amortizing the total initial investment, which includes the membrane unit cost $P_{m}$ (548 USD/m^2^) and fixed ancillary costs $P_{mf}$ (822 USD/m^2^) multiplied by the area $A_{m}$, over a 10-year depreciation period, research has confirmed that its unique MFI composite structure retains its initial permeation and separation performance even after 10 years of intermittent operation [47,48], demonstrating its long-term stability. The typical service life expectancy for high-performance membranes is generally 5 to 10 years.

The fixed costs for the vacuum pump and expander are calculated using the following equations [46]:

(8) $$C_{2}=\frac{500\times \sum P_{i}}{25\times 24\times 365}$$

In the equation, the compressor cost follows a benchmark of 500 USD/kW of compression power demand. Here, $C_{2}$ represents the compressor cost in USD/h, and $\sum P_{i}$ denotes the power of the compressor. The hourly cost is calculated based on an assumed equipment service life of 25 years.

The annual operating time of the process is estimated at 8000 h, with an average electricity price e of 0.14 USD/kWh. The operating cost of the overall process is calculated as follows [46]:

(9) $$C_{3}=\left(\sum P_{i}-P_{\mathrm{expander}}\right)\times e$$

In the equation, $C_{3}$ represents the total electricity cost of the process, e denotes the average electricity price, taken as 0.14 USD/kWh in this study, and $\sum P_{i}$ refers to the total energy consumption of the compressors in the process.

In addition to operating costs, installation and maintenance costs are also considered. Typically, installation cost is estimated at 60% of the total major capital cost of the system, which also includes minor capital costs such as those for piping and instrumentation. Therefore, assuming a membrane system service life of 25 years, the hourly depreciation rate is calculated as follows [46]:

(10) $$C_{4}=0.6\times \left(C_{1}+C_{2}\right)\times \frac{5}{25}$$

Maintenance and labor costs are typically assumed to be 10% of the total capital cost [46]:

(11) $$C_{5}=0.1\times \left(C_{1}+C_{2}+C_{4}\right)$$

The total cost is calculated as follows C.

(12) $$C=C_{1}+C_{2}+C_{3}+C_{4}+C_{5}$$

The capital cost of the distillation column is divided into two components: the column shell and trays cost, and the cost of the reboiler and condenser. The cost of the column shell and trays is calculated using the following equation:

The costs of the reboiler and condenser are calculated as follows [49]:

(13) $$C_{BM}=C_{p}\times F_{BM}$$

(14) $$\log_{10}C_{p}^{0}=K_{1}+K_{2}\log_{10}(A)+K_{3}{\left[\log_{10}(A)\right]}^{2}$$

(15) $$F_{BM}=B_{1}+B_{2}\times F_{M}\times F_{P}$$

In the equation, $C_{BM}$ represents the bare module cost, $C_{p}$ denotes the purchased cost in 2019, and $F_{BM}$ refers to the bare module factor. (The specific values of parameters K, $B_{1}$, $B_{2}$, $F_{M}$, and $F_{P}$, along with detailed calculation procedures, are provided in the. Analysis, Synthesis and Design of Processes (Fourth Edition 2012), 530 pages

The Capital Recovery Factor (CRF) is used to convert a one-time capital investment (calculated as the bare module cost) into an equivalent annualized cost, thereby accounting for the time value of money [49].

(16) $$CRF=\frac{i{\left(1+i\right)}^{n}}{{\left(1+i\right)}^{n}-1}$$

In the equation, CRF represents the capital recovery factor, i denotes the discount rate, which is set at 10% in this study, and n refers to the equipment lifespan, taken as 15 years.

The total bare module cost (year 2019 basis) comprises the costs of the column system and the heat exchanger system [50]. The annualized total cost is calculated as the product of the total bare module cost and the capital recovery factor (CRF).

## 3. Results and Discussion

### 3.1. Distillation—Full Recovery Process for Membrane Products Part One: Distillation

The distillation column serves as a key unit operation in the propane-butane separation process, designed to recover high-purity propane and butane from a hydrocarbon mixture. Product quality directly influences the economic value of liquefied petroleum gas (LPG), necessitating strict adherence to specified purity levels. Rigorous process control is essential to optimize the trade-off between maximizing production capacity and minimizing energy consumption, while ensuring operational safety and compliance with environmental regulations. Given the high propane content in the feedstock and its stable molecular structure, propane is widely utilized in several high-value applications. These include eco-friendly refrigerants, marine engine fuels, the green synthesis of propylene, and its selective one-step conversion to acetone via the heterogeneous electro-Fenton process (h-EFP). The corresponding purity requirements for these applications are high concentration, 100%, 99.95%, and 99.9%, respectively. To meet industrial standards, the propane stream leaving the top of the distillation column must therefore maintain a purity of at least 99%. Accordingly, in the present study, the purity of the overhead propane product is set at 99%.

In the economic analysis, the operating costs of the distillation column primarily consist of energy consumption and equipment-related expenses. The energy costs include expenditures for cooling water in the condenser and steam in the reboiler, with an annual operating time of 8000 h. These are calculated as 32,920.65 USD/year and 234,723.26 USD/year, respectively, resulting in a total energy cost of 267,643.91 USD/year. The equipment-related cost comprises the annualized capital investment of the distillation system (including the reboiler, condenser, and column shell), which amounts to 149,059.62 USD/year.

### 3.2. Membrane Flow Design for the Full Product Collection Distillation—Membrane Separation Process

Based on the broad application requirements of propane as both fuel and chemical feedstock, this process sets the propane product purity at 99% during the distillation stage to meet industrial-grade quality standards. The distillation column is fed with a liquid mixture at a flow rate of 1933 kg/h, consisting of 70% propane, 21% isobutane, and 9% n-butane. Operating under 14 bar with a top temperature of 120 °C, the column achieves preliminary separation of propane and butane: a 99%-purity propane stream is obtained overhead, while the bottoms yield a butane-enriched stream (total butane content ≈ 99%), which is used as feed for the subsequent separation of n-/isobutane. This butane-enriched stream is further sent to a two-stage membrane system for efficient separation of n-butane and isobutane. To systematically evaluate the separation performance and economic potential of different membrane process configurations, three process figures were designed for comparison, including one two-stage configuration without recycling and three two-stage configurations incorporating different recycle strategies.

Based on material balance results, the distillation column produces a propane product stream with a flow rate of 30.96 kmol/h and a butane-enriched stream of 9.79 kmol/h. This butane-rich stream, serving as the feed to the two-stage membrane separation system, has a total mass flow rate of 569.3 kg/h under operating conditions of 14 bar and 80 °C. Its composition consists of 170.8 kg/h n-butane and 398.5 kg/h isobutane, corresponding to an n-butane mole fraction of approximately 30%.

**Figure 2:**
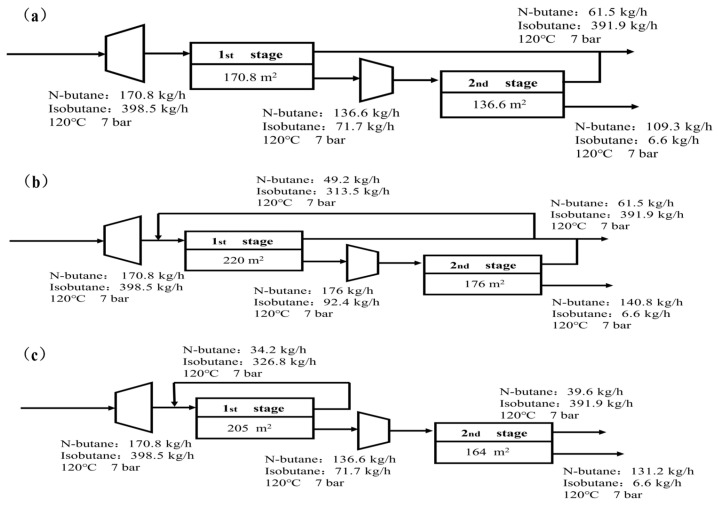
Flowchart of secondary membrane separation: (**a**) two-stage non-recycle membrane process; (**b**) two-stage membrane process with 80% post-stage permeate reflux; (**c**) two-stage pre- reflux with full recirculation.

A conventional two-stage membrane separation process typically employs a continuous configuration, where the addition of a second membrane unit enhances product purity to meet target specifications. Accordingly, a two-stage continuous membrane system was designed to achieve comprehensive utilization of the products. As illustrated in Figure 2a, a two-stage non-recycle membrane process is adopted for n-/isobutane separation. Under the process target of achieving 80% n-butane recovery, simulation and optimization results indicate that the required membrane area is 170.8 m^2^ in the first stage and 136.6 m^2^ in the second stage. Cost analysis based on this configuration shows that the membrane-related operating cost is 4.85 USD/h, while the total operating cost of the system reaches 11.66 USD/h. Based on these values, the total separation cost per kilogram of butane product is calculated to be 0.047 USD/kg.

Merely increasing the number of membrane modules to enhance product purity would significantly raise process costs. Therefore, a reflux configuration is introduced, where a portion of the permeate gas is recycled to improve both product purity and yield. As shown in Figure 2b, a two-stage membrane process with 80% post-stage permeate reflux is employed for n-/isobutane separation. Under the target of achieving 80% n-butane recovery, process simulation and optimization indicate that the required membrane area is 220 m^2^ in the first stage and 176 m^2^ in the second stage. Cost analysis based on this configuration shows that the membrane-related operating cost is 6.26 USD/h, and the total operating cost amounts to 14.57 USD/h. The corresponding specific separation cost per kilogram of butane product is calculated to be 0.046 USD/kg.

Following the study of partial post-stage reflux, a configuration with full pre-stage permeate reflux was further investigated. As illustrated in Figure 2c, the process employs a two-stage membrane system with 80% full pre-stage permeate reflux for n-/isobutane separation. Under the target n-butane recovery rate of 80%, process simulation and optimization indicate that the required membrane area is 205 m^2^ in the first stage and 164 m^2^ in the second stage. Cost analysis shows a membrane-related operating cost of 5.82 USD/h and a total operating cost of 13.67 USD/h for this configuration, resulting in a specific separation cost of 0.047 USD/kg of butane product.

In the comparative analysis of the three membrane processes, the pre-stage and post-stage reflux configurations show no significant difference in energy consumption per unit product, indicating similar energy efficiency. However, from the perspective of system integration and long-term operational stability, the post-stage reflux configuration demonstrates distinct engineering advantages: it operates with a lower gas flux through the membrane modules. The cost data for each stage of the membrane treatment process across the three procedures is presented in Table 1. This characteristic not only helps mitigate membrane fouling and extend service life but also reduces operating costs and maintenance requirements associated with frequent module replacement.

Based on integrated technical and economic analysis, the partial reflux configuration demonstrates the most favorable performance among the three reflux strategies. This is because the unit cost of the partial recirculation configuration is the lowest among the three options, at 0.045 USD/h. It maintains relatively low energy consumption while achieving lower unit product cost, offering a more competitive and economically viable technological pathway for the long-term operation of industrial-scale distillation-membrane hybrid systems. The cost data for the two stages within the entire process is presented in Table 2.

### 3.3. Analysis of Processes Related to Membrane Performance

In the distillation-membrane hybrid separation process, the membrane module serves as the core unit for achieving high-selectivity separation, with its performance directly determining the separation efficiency, energy consumption, and economic viability of the entire system. Optimizing the design of membrane components is essential for process intensification, wherein membrane area, separation factor, and permeation flux represent the key parameters governing its performance and techno-economic characteristics.

Membrane area serves as the fundamental parameter governing processing capacity and equipment scale, directly influencing the driving force for mass transfer and the extent of separation. Insufficient area limits permeation flux and recovery rate, while excessive area increases capital and operating costs, and may lead to issues such as uneven flow distribution. The separation factor, a thermodynamic parameter reflecting the intrinsic selectivity of the membrane material, indicates its ability to differentiate between the permeation rates of different components. A higher separation factor enhances product purity, reduces downstream purification load, and may decrease the required membrane area and number of process stages. Permeation flux, as a key kinetic indicator, affects separation efficiency and equipment size, but often exhibits a trade-off relationship with the separation factor, necessitating a balance between material selection and process parameters.

**Figure 3:**
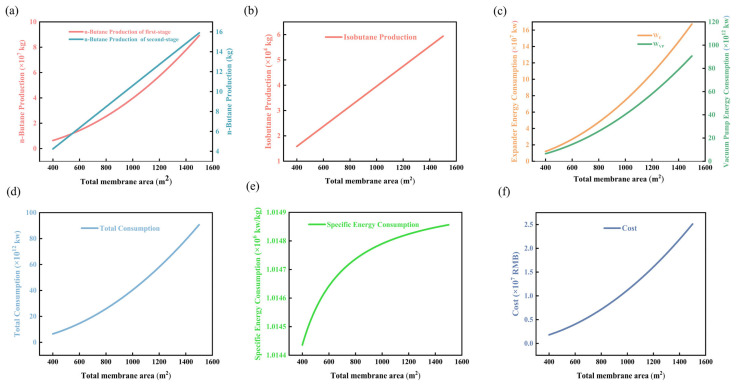
Diagram of the relationship between membrane area changes: (**a**) n-butane production chart; (**b**) isobutane production chart; (**c**) energy consumption chart for vacuum pumps and compressors; (**d**) total energy consumption chart; (**e**) specific energy consumption chart; (**f**) total cost chart.

Therefore, this study sets the membrane area at 400–1500 m^2^, n-butane permeance at 0.6–1.2 kg·m^−2^·h^−1^, and the separation factor at 10–65. As shown in Figure 3 [51], as the membrane area increases from 400 to 1600 m^2^, the production rates of n-butane and isobutane in the first stage increase simultaneously but at a decelerating rate, while the n-butane output in the second stage is approximately 15–20% lower due to reduced concentration. The curves for all three stages converge beyond 1200 m^2^, with marginal increments below 5%.

As shown in Figure 3d–f, Specific energy consumption exhibits a U-shaped trend, with a minimum of 1.0145 kWh·kg^−1^ corresponding to an area of 800–1000 m^2^. Total energy consumption increases linearly with a slope of 0.42 kWh·m^−2^ and an intercept of 28 kWh, representing the base load. The overall cost reaches a minimum of 1.0144–1.0146 USD·kg^−1^ in the range of 1000–1200 m^2^. Beyond 1400 m^2^, membrane replacement costs increase by 3.8%, and the economically optimal region coincides with the energy consumption optimum.

**Figure 4:**
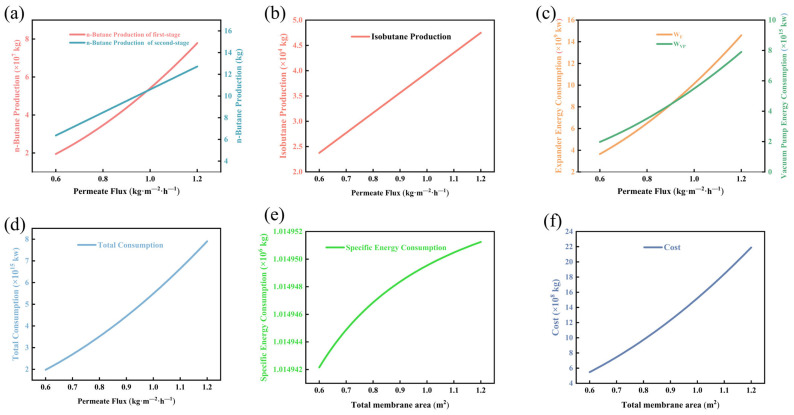
Relationship diagram of permeation flux variation: (**a**) n-butane production chart; (**b**) isobutane production chart; (**c**) energy consumption chart for vacuum pumps and compressors; (**d**) total energy consumption chart; (**e**) specific energy consumption chart; (**f**) total cost chart.

As shown in Figure 4, when the permeation flux (J) increases from 0.6 to 1.2 kg·m^−2^·h^−1^, Figure 4 [51] illustrate its effect on the production rates in the two-stage membrane separation process. Within the range of J = 0.6–1.2 kg·m^−2^·h^−1^, the n-butane production rate in the first stage increases linearly from 5.0 kg·h^−1^ to 14 kg·h^−1^, while that in the second stage rises from 4.0 kg·h^−1^ to 12 kg·h^−1^, with a slope reduction of approximately 15%, reflecting the gradual decline in mass transfer driving force due to concentration polarization. The isobutane production rate remains 10–15% lower overall, confirming the high selectivity of the membrane toward linear alkanes. When J ≥ 1.0 kg·m^−2^·h^−1^, all three curves approach asymptotic limits, with marginal production gains below 3%, indicating that the flux is nearing the intrinsic permeation limit of the membrane. In Figure 4d, the specific energy consumption (SEC) decreases monotonically with increasing J, from 1.014952 kWh·kg^−1^ to 1.014942 kWh·kg^−1^, a reduction of 0.98%. The trend follows a power–law relationship SEC ∝ J^−0.9^ (R^2^ > 0.99), suggesting that the dilution effect resulting from higher flux dominates the reduction in compression work. Figure 4e shows a linear negative correlation between total energy consumption and J, with a slope of −18 kWh per unit increases in kg·m^−2^·h^−1^ and an intercept of 22 kWh, reflecting the concurrent decrease in pump circulation and cooling load under high-flux conditions. Figure 4f integrates capital and operating costs, revealing an inflection point in the cost curve at J = 1.0 kg·m^−2^·h^−1^. Beyond this point, further increases in flux yield cost reductions of less than 0.2%, while the associated rise in membrane replacement frequency due to fouling offsets energy-saving benefits. Therefore, for industrial scale-up, it is recommended to maintain the permeation flux at 1.0 ± 0.05 kg·m^−2^·h^−1^ to achieve an optimal balance among production rate, energy consumption, and membrane lifespan.

**Figure 5:**
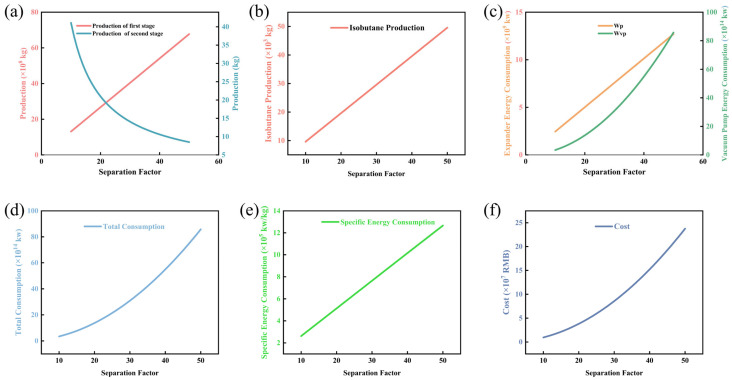
Diagram of the relationship between changes in separation factors: (**a**) n-butane production chart; (**b**) isobutane production chart; (**c**) energy consumption chart for vacuum pumps and compressors; (**d**) total energy consumption chart; (**e**) specific energy consumption chart; (**f**) total cost chart.

Figure 5 illustrates the influence of the separation factor (α) within the range of 10–65 on system performance. As α increases, the production rates of n-butane and isobutane rise simultaneously, from 4 × 10^4^ kg/h to 8 × 10^4^ kg/h and from 2 × 10^4^ kg/h to 6 × 10^4^ kg/h, respectively, indicating that higher selectivity enhances component recovery. This phenomenon can be attributed to the enhanced selectivity of the separation process at higher α values, which effectively reduces the mutual entrainment of the two components, thereby promoting the efficient separation and recovery of target products. However, this is accompanied by a sharp increase in vacuum pump energy consumption, which rises from approximately 5 × 10^6^ J/h to 15 × 10^6^ J/h, driving the total energy consumption from 7 × 10^6^ J/h to 17 × 10^6^ J/h and representing a major energy bottleneck. Notably, the specific energy consumption (energy consumption per unit mass of product) of the system exhibits a shallow U-shaped variation trend with the increase in α, reaching a minimum of 0.8 × 10^6^ J/kg at α ≈ 40. In contrast, the total cost increases linearly with α, rising from 2 × 10^5^ USD to 4.5 × 10^5^ USD. Considering production, energy use, and cost together, the optimal operating point lies at α ≈ 40, where production is significantly improved and specific energy consumption is minimized. Although further increasing α beyond this point may yield approximately 10% additional production gains, it would lead to 30% and 25% increases in energy consumption and cost, respectively, resulting in markedly diminished economic returns. Therefore, it is recommended to maintain α within the range of 35–45 to achieve the best balance between recovery efficiency and process economics.

This section systematically investigates the synergistic effects of membrane area, permeation flux, and separation factors on the performance of the distillation-membrane hybrid process. The results indicate that within the membrane area range of 400–1500 m^2^, both total energy consumption and cost exhibit a nonlinear increase, with the growth rate accelerating significantly beyond 800 m^2^. Increasing the permeation flux within the range of 0.6–1.2 kg·m^−2^·h^−1^ enhances product output but is accompanied by an exponential rise in vacuum pump energy consumption and a distinct minimum in specific energy consumption at J ≈ 0.9 kg·m^−2^·h^−1^. Enhancing the separation factor within the range of 10–65 improves component recovery yet leads to a sharp increase in energy consumption and cost, with the minimum specific energy consumption observed at α ≈ 40, representing the most economically favorable operating point.

In summary, to achieve optimal process economics, it is recommended to maintain the membrane area below approximately 800 m^2^, set the permeation flux at 0.9 kg·m^−2^·h^−1^, and select a separation factor between 35 and 45. This strategy ensures high separation efficiency while effectively controlling energy consumption and operating costs.

### 3.4. Analysis of Operating Conditions Related to Membrane Processes

The key operating conditions in the overall process are primarily the pressure and temperature at the expander outlet, which also correspond to the conditions under which the stream enters the MFI hollow fiber membrane for separation. Therefore, a further investigation was conducted on the effects of temperature and pressure, with the studied ranges set at 373.15–403.15 K and 5–8 bar, respectively.

**Figure 6:**
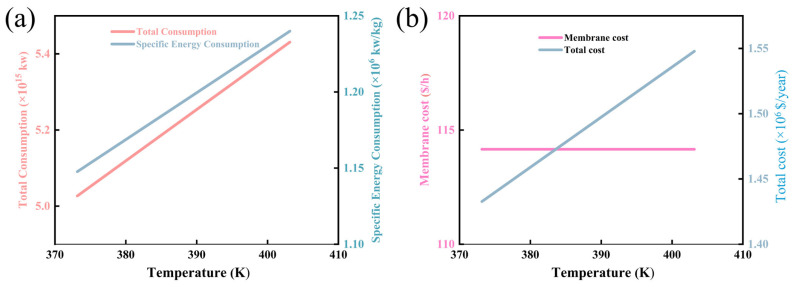
Chart of changes in process cost, energy consumption and specific energy consumption affected by temperature: (**a**) temperature versus total energy consumption and specific energy consumption diagram; (**b**) temperature versus membrane cost and total cost relationship diagram.

Figure 6a,b illustrate the influence of temperature on the energy consumption and cost of the membrane separation process. As presented in Figure 6a, within the investigated temperature range of 370 K to 410 K, both the total energy consumption (pink curve) and the specific energy consumption (energy consumption per unit mass of permeate, blue curve) exhibit a monotonic linear growth trend. Specifically, the total energy consumption increases from approximately 5.0 × 10^5^ J/h to 5.4 × 10^6^ J/h, with a net increase of 0.4 × 10^6^ J/h and a relative growth rate of 8%. Concurrently, the specific energy consumption rises from 1.10 × 10^6^ J/kg to 1.25 × 10^6^ J/kg, corresponding to a relative increment of 13.6%. From a physical perspective, this trend originates from the intrinsic dependence of membrane mass transfer resistance on temperature. Although elevated temperature enhances the thermal motion of permeating molecules (as described by the Arrhenius equation, where molecular diffusivity increases with temperature), it simultaneously induces subtle structural changes in the membrane matrix. For most polymer-based separation membranes, moderate temperature elevation (within 370–410 K) can lead to the densification of the membrane’s selective layer or the reduction in free volume in the polymer chains. This structural change increases the steric hindrance for molecular permeation, thereby elevating the overall permeation resistance of the membrane. To overcome this enhanced resistance and maintain the desired permeation flux (a key performance indicator for industrial membrane separation processes), the system must input additional energy to drive the mass transfer process—manifested as the observed increase in total and specific energy consumption.

Figure 6b further reveals the effect of temperature on process costs, distinguishing between membrane cost (pink curve) and total process cost (blue curve). Notably, the membrane cost remains essentially stable with increasing temperature, showing no significant fluctuation throughout the 370–410 K range. In contrast, the total process cost (blue curve) increases linearly from 1.40 × 10^6^ USD/year to 1.55 × 10^6^ USD/year, with a total increase of 1.5 × 10^5^ USD/year and a relative growth rate of 10.7%. This cost variation pattern directly reflects the dominant contribution of energy consumption to the total process cost under the investigated temperature conditions.

**Figure 7:**
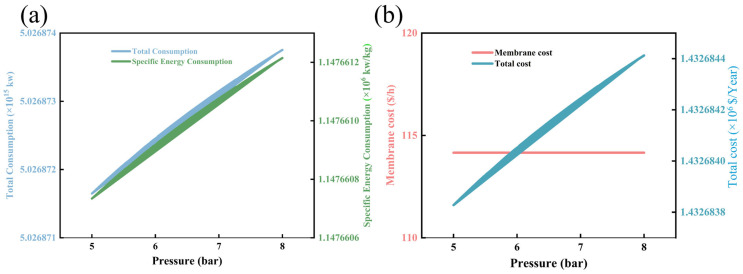
Graph of changes in process cost, energy consumption and specific energy consumption affected by pressure: (**a**) pressure versus total energy consumption and specific energy consumption diagram; (**b**) pressure versus membrane cost and total cost relationship diagram.

Figure 7a,b analyze the influence of pressure on energy consumption and cost in the membrane separation process. As illustrated in Figure 7a, within the investigated operating pressure range of 5 bar to 8 bar, both the total energy consumption (blue curve) and the specific energy consumption (energy consumption per unit mass of permeate, green curve) exhibit a monotonic linear upward trend, albeit with relatively small absolute increments. Specifically, the total energy consumption increases slightly from 5,053,871 J/h to 5,426,874 J/h, corresponding to a net increment of merely 3.7 × 10^5^ J/h and a relative growth rate of 7.38%. Concurrently, the specific energy consumption rises from 11,476,606 J/kg to 12,326,612 J/kg, with a net increase of 8.5 × 10^5^ J/kg and a relative growth rate of 7.41%. From the perspective of physical and mass transport mechanisms, this subtle yet consistent upward trend of energy consumption with increasing pressure can be attributed to two interrelated factors. First, while an increase in operating pressure enhances the transmembrane pressure difference (the primary driving force for molecular permeation), it also induces slight compaction of the membrane selective layer. This enhanced permeation resistance necessitates a marginal increase in energy input to maintain the target permeation flux, as the system must overcome both the elevated membrane resistance and the higher fluid dynamic resistance of the feed stream under increased pressure. Second, the compression and transportation of the feed gas/liquid stream to the higher operating pressure require additional energy input from the compression unit, which contributes to the linear growth of total energy consumption. The extremely small magnitude of the energy increment indicates that the membrane system exhibits excellent pressure stability within the studied range, and the pressure-induced membrane compaction and increment in fluid resistance are relatively insignificant.

Figure 7b further clarifies the effect of operating pressure on the economic cost structure of the process, distinguishing between membrane cost (red curve) and total process cost (blue curve). Notably, the membrane cost remains essentially constant with the variation in operating pressure, showing no detectable fluctuation throughout the 5–8 bar range. In contrast, the total process cost (blue curve) increases linearly from 14,326,834 USD/year to 15,426,838 USD/year, with a net increment of 1.1 × 10^6^ USD/year and a relative growth rate of 8.1%. This cost variation pattern directly reflects that the slight increase in total cost is dominated by the elevated energy consumption, while the membrane cost is insensitive to operating pressure within the studied range. The stability of membrane cost can be explained by the physical and chemical stability of the membrane material under moderate pressure conditions: the operating pressure range of 5–8 bar is far below the mechanical failure threshold of the membrane material and module, and does not induce degradation of the membrane’s chemical structure or shorten its service life. Consequently, the core factors determining membrane cost—including membrane material cost, fabrication process cost, and module replacement frequency—remain unchanged. This observation confirms that membrane cost is primarily governed by intrinsic material properties and manufacturing technologies rather than short-term fluctuations in operating pressure within the membrane’s stable service range.

In the operational parameter study, the effects of expander outlet temperature (373.15–403.15 K) and pressure (5–8 bar) on system performance were investigated. The results indicate that both increases in temperature within the range of 373.15–403.15 K and increases in pressure within the range of 5–8 bar lead to a linear rise in total costs, total energy consumption, and energy consumption per unit. However, the overall relative increases were modest: a 10.7% rise in temperature-related costs and an 8% increase in energy consumption, alongside an 8.1% increase in pressure-related costs and a 7.38% rise in energy consumption. This implies that under the current operating conditions, variations in temperature and pressure exert a relatively minor influence on costs and energy consumption.

## 4. Conclusions

In the process design and techno-economic analysis of the distillation-membrane hybrid separation system, the two-stage membrane process with a “partial residue-side reflux” configuration was found to achieve a total operating cost of 31.58 USD/h, demonstrating favorable economic performance. Building on this, a parameter optimization study was further conducted on the membrane separation unit to identify key operational and structural parameters influencing system performance. Through systematic simulation and sensitivity analysis, it was determined that a membrane area of 800 m^2^, a permeation flux of 0.9 kg·m^−2^·h^−1^, and a separation factor of 40 collectively represent the optimal configuration. Under these conditions, the system achieves an optimal balance between separation efficiency and operating cost, enabling high n-alkane recovery while effectively controlling energy consumption and capital investment. This configuration is identified as the most favorable process setup for the hybrid system.

## Figures and Tables

**Table 1:** Cost data of each process in the membrane process.

Process	Fixed Cost (USD/h)	Installation Depreciation (USD/h)	Operating Cost (USD/h)	Maintenance Cost (USD/h)	Total Cost (USD/h)	Unit Cost (USD/h)
Two-stage series connection	4.85	1.25	1.14	4.39	11.66	0.047
Partial reflux	6.26	1.62	1.03	5.65	14.57	0.046
Pre-reflux	5.83	1.50	1.05	5.27	13.67	0.047

**Table 2:** Cost data for two stages within the entire process and total cost figures.

	Distillation Section	Membrane Section	General Process
Cost	17.02 USD/h	14.57 USD/h	31.58 USD/h

## Data Availability

The raw data supporting the conclusions of this article will be made available by the authors on request.

## References

[1] Vasudevan H., Zolghadri S., Makarem M.A., Rahimpour M.R., Omidvar B., Shirazi N.A., Makarem M.A. (2023). 2—Introduction to oil, gas, and petrochemical industries: Importance to the current world. Crises in Oil, Gas and Petrochemical Industries.

[2] Ajuka L.O., Kazeem R.A., Kuti O.A., Jen T.C., Afolalu A.S., Akinlabi E.T. (2024). Decarbonized automotive fuel: Liquefied petroleum gas biosynthesis, benefits and drawbacks. Results Eng..

[3] Sugie H., Sasaki C., Hashimoto C., Takeshita H., Nagai T., Nakamura S., Furukawa M., Nishikawa T., Kurihara K. (2004). Three cases of sudden death due to butane or propane gas inhalation: Analysis of tissues for gas components. Forensic Sci. Int..

[4] Jenck J.F., Agterberg F., Droescher M.J. (2004). Products and processes for a sustainable chemical industry: A review of achievements and prospects. Green Chem..

[5] Ekejiuba A. (2021). Natural Petroleum: Chemistry and Valuable Products Fractions. Carbon.

[6] Ajayi O.O., Banjo S.O., Bolaji B.O., Oyelaran O.A.A., Kazi M.S.N. (2021). Impact of Working Fluids and Performance of Isobutane in the Refrigeration System. Low-Temperature Technologies and Applications.

[7] Rahman M., Kabir S., Haque A.K.M. (2018). Occurrence, distribution, and origin of shallow biogenic gas in late quaternary unconsolidated sand deposit of shahbazpur structure, Southern Bangladesh. Pet. Coal.

[8] Liu H., Chen H., Xiao Q., Fang X., Tang Z. (2021). More on Sombor indices of chemical graphs and their applications to the boiling point of benzenoid hydrocarbons. Int. J. Quantum Chem..

[9] Zhang Z., Fratantonio D., Barrot Lattes C., Rojas-Cardenas M., Colin S. (2024). Measurements of diffusion coefficient and kinetic diameter of acetone vapor via molecular tagging. Microfluid. Nanofluidics.

[10] Motalov V.B., Korobov M.A., Dunaev A.M., Dunaeva V.V., Kudin L.S. (2023). Saturated Vapor Pressure and Sublimation Enthalpy of l-serine and l-cysteine. J. Chem. Eng. Data.

[11] Herzog C.D. (2021). Develop Distillation Controls During Process Design. Chem. Eng. Prog..

[12] Yang L., Chen Y., Wang J., Luo Y., Zhou P., Zhang X. (2024). The Simulation and Optimization of the Tetrafluoroethylene Rectification Process. Separations.

[13] Zhou J., Lv P., He B., Wu J., Wang G., Ma H., Wang Y., Chen G. (2024). Optimisation of the Ethanol Fermentation Process Using Hydrothermal Pretreatment of Cellulose Waste-Effect of Fermentation Pattern and Strain. Molecules.

[14] Malaguti M., Presson L.K., Tiraferri A., Hickenbottom K.L., Achilli A. (2024). Productivity, selectivity, and energy consumption of pilot-scale vacuum assisted air-gap membrane distillation for the desalination of high-salinity streams. Desalination.

[15] Kooijman H.A., Sorensen E. (2022). Recent advances and future perspectives on more sustainable and energy efficient distillation processes. Chem. Eng. Res. Des..

[16] He Y., Zhang B., Zhou S., Wei Y., Li B., Wang H. (2025). Stepwise Removal Mechanism of Impurity Using Multi-Flux During Blister Copper Pyrometallurgical Refining. Metall. Mater. Trans. B.

[17] Wang H., Song S., Zhang Z., Xin L., Wang T., Wang L. (2022). A novel process of low-temperature fractionation combined with extractive distillation for H_2_S removal from natural gas. Sep. Purif. Technol..

[18] Klein H., Fritsch P., Haider P., Kender R., Rößler F., Rehfeldt S., Freko P., Hoffmann R., Thomas I., Wunderlich B. (2021). Flexible Operation of Air Separation Units. ChemBioEng Rev..

[19] Mathew T.J., Narayanan S., Jalan A., Matthews L., Gupta H., Billimoria R., Pereira C.S., Goheen C., Tawarmalani M., Agrawal R. (2022). Advances in distillation: Significant reductions in energy consumption and carbon dioxide emissions for crude oil separation. Joule.

[20] Yang Q., Xu W., Li J., Wang Z., Xu H., Zhou M., Wang Y., Li X., Zhong L., Cui P. (2024). Molecular mechanism of efficient separation of isopropyl alcohol and isooctane by extractive distillation. Chem. Eng. Res. Des..

[21] Parliment T.H. (2020). Solvent extraction and distillation techniques. Techniques for Analyzing Food Aroma.

[22] Yu R., Liu S., Wang X. (2024). Dataset Distillation: A Comprehensive Review. IEEE Trans. Pattern Anal. Mach. Intell..

[23] Jiang T.-H., Gong Y., Yang Z.-G. (2023). Failure analysis on abnormal damage and corrosion of methanol distillation column in MTBE production plant. Int. J. Press. Vessel. Pip..

[24] Demirel S.E., Li J., Hasan M.M.F. (2021). Membrane Separation Process Design and Intensification. Ind. Eng. Chem. Res..

[25] Knebel A., Caro J. (2022). Metal–organic frameworks and covalent organic frameworks as disruptive membrane materials for energy-efficient gas separation. Nat. Nanotechnol..

[26] Teodoro J.A., Arend G.D., Proner M.C., Verruck S., Rezzadori K. (2023). A review on membrane separation processes focusing on food industry environment-friendly processes. Crit. Rev. Food Sci. Nutr..

[27] Asadi J., Kazempoor P. (2022). Dynamic response and flexibility analyses of a membrane-based CO_2_ separation module. Int. J. Greenh. Gas Control.

[28] Liu Y., Zhao G., Tong F., Li Z., Wang L., Fang C., Lei L., Xu Z. (2025). Sandwich-like multilayer hollow fiber carbon membranes for gas separations. J. Membr. Sci..

[29] Fu J., Luo H., Wang S., Jin X., Yang Z., Gao X., Gu X. (2025). Optimization of internal coupling process of NaA zeolite membrane in distillation column to separate ethanol/water mixture. J. Membr. Sci..

[30] Liu H., Liang L., Tian F., Xi X., Zhang Y., Zhang P., Cao X., Bai Y., Zhang C., Dong L. (2024). Scalable Preparation of Ultraselective and Highly Permeable Fully Aromatic Polyamide Nanofiltration Membranes for Antibiotic Desalination. Angew. Chem. Int. Ed. Engl..

[31] Rom A., Miltner A., Wukovits W., Friedl A. (2016). Energy saving potential of hybrid membrane and distillation process in butanol purification: Experiments, modelling and simulation. Chem. Eng. Process. Process Intensif..

[32] Wang Z., Xu J., Pati S., Chen T., Deng Y., Dewangan N., Meng L., Lin J.Y.S., Kawi S. (2020). High H_2_ permeable SAPO-34 hollow fiber membrane for high temperature propane dehydrogenation application. AIChE J..

[33] Wu J., Wu H., Wang B., Zhou R., Xing W. (2022). One-Step Scalable Fabrication of Highly Selective Monolithic Zeolite MFI Membranes for Efficient Butane Isomer Separation. ACS Appl. Mater. Interfaces.

[34] Vroon Z.A.E.P., Keizer K., Gilde M.J., Verweij H., Burggraaf A.J. (1996). Transport properties of alkanes through ceramic thin zeolite MFI membranes. J. Membr. Sci..

[35] Bakker W.J.W., Kapteijn F., Poppe J., Moulijn J.A. (1996). Permeation characteristics of a metal-supported silicalite-1 zeolite membrane. J. Membr. Sci..

[36] Liu Y., Li M., Chen Z., Cui Y., Lu J., Liu Y. (2021). Hierarchy Control of MFI Zeolite Membrane towards Superior Butane Isomer Separation Performance. Angew. Chem. Int. Ed..

[37] Saulat H., Song W., Yang J., Yan T., He G., Tsapatsis M. (2022). Fabrication of b-oriented MFI membranes from MFI nanosheet layers by ammonium sulfate modifier for the separation of butane isomers. J. Membr. Sci..

[38] Liu Y., Chen S., Ji T., Yan J., Ding K., Meng S., Lu J., Liu Y. (2023). Room-Temperature Synthesis of Zeolite Membranes toward Optimized Microstructure and Enhanced Butane Isomer Separation Performance. J. Am. Chem. Soc..

[39] Voß H., Diefenbacher A., Schuch G., Richter H., Voigt I., Noack M., Caro J. (2009). Butene isomers separation on titania supported MFI membranes at conditions relevant for practice. J. Membr. Sci..

[40] Lee T., Choi J., Tsapatsis M. (2013). On the performance of c-oriented MFI zeolite Membranes treated by rapid thermal processing. J. Membr. Sci..

[41] Ma B., Zhu Y., Hong H., Cui L., Gao H., Zhao D., Wang B., Zhou R., Xing W. (2022). Improved silicalite-1 membranes on 61-channel monolithic supports for n-butane/i-butane separation. Sep. Purif. Technol..

[42] Choi J., Ghosh S., King L., Tsapatsis M. (2006). MFI zeolite membranes from a- and randomly oriented monolayers. Adsorption.

[43] Li Q., Wu H., Wang Z., Wang J. (2022). Analysis and optimal design of membrane processes for flue gas CO_2_ capture. Sep. Purif. Technol..

[44] Feng X., He C. (2007). Chemical Engineering Principles.

[45] Ayazi A., Babakhani D., Soleymani M. (2022). Simulation of a Depropanizer Distillation Column and Validation of Results by Industrial Data. Chem. Eng. Technol..

[46] Shao P., Dal-Cin M.M., Guiver M.D., Kumar A. (2013). Simulation of membrane-based CO_2_ capture in a coal-fired power plant. J. Membr. Sci..

[47] Yu K., Fang J., Wu H., Zhou R., Xu N. (2026). Mild synthesis of inside tubular MFI zeolite membranes in gel-recycling mode for efficient butane isomer separation. J. Membr. Sci..

[48] Deng Z., Nicolas C.H., Daramola M.O., Sublet J., Schiestel T., Burger A.J., Guo Y., Giroir-Fendler A., Pera-Titus M. (2010). Nanocomposite MFI-alumina hollow fibre membranes prepared via pore-plugging synthesis: Influence of the porous structure of hollow fibres on the gas/vapour separation performance. J. Membr. Sci..

[49] Turton R., Bailie R.C., Whiting W.B., Shaeiwitz J.A. (2008). Analysis, Synthesis and Design of Chemical Processes.

[50] Ferrario D., Pröll T., Stendardo S., Lanzini A. (2024). Cost and environmentally efficient design of an absorption-based post-combustion carbon capture unit for industry applications. Chem. Eng. J..

[51] Garea S.-A., Corbu A.-C., Deleanu C., Iovu H. (2006). Determination of the epoxide equivalent weight (EEW) of epoxy resins with different chemical structure and functionality using GPC and 1H-NMR. Polym. Test..

